# Arterial Stiffness Changes in Adult Cancer Patients Receiving Anticancer Chemotherapy: A Real-World Bicentric Experience

**DOI:** 10.7759/cureus.56647

**Published:** 2024-03-21

**Authors:** Salim Benkhedda, Nacera Bengherbi, Yahia Cherifi, Souhila Ouabdesselam, Nabila Waheed, Clara M Harris

**Affiliations:** 1 Cardiology, Cardiology Oncology Collaborative Research Group, Faculty of Family Medicine, University of Algiers Benyoucef Benkhedda, Algiers, DZA; 2 Radiation Oncology, The Center for Cancer & Blood Disorders, Fort Worth, USA; 3 Internal Medicine, Baylor Scott & White All Saints Medical Center – Fort Worth, Fort Worth, USA

**Keywords:** arterial stiffness, cancer patients, post-chemotherapy cardiotoxicity, pulse wave velocity, chemotherapy agents

## Abstract

Background

Chemotherapy correlates to acute and long-term cardiotoxicity, is reflected clinically by myocardial and vascular endothelial dysfunction, and can cause cardiovascular complications. Thus, early diagnosis of cardiovascular disease in cancer patients undergoing anti-cancer treatment is necessary to enhance long-term survival. Our principal objective in this study was to discern the impact of specific anti-cancer chemotherapeutics and biologics on arterial stiffness alterations before and after the administration.

Methods

Conducted at Mustafa Bacha University Hospital, Algeria, the study focused on arterial stiffness in anti-cancer chemotherapy patients. Assessments included blood pressure, diabetes, and dyslipidemia, with precise measurements using validated systems, particularly pulse wave velocity (PWV). Various chemotherapy protocols were applied, and statistical analysis with R software (R Foundation for Statistical Computing, Vienna, Austria) maintained a significance level of p=0.05. Key outcomes centered on carotid-femoral PWV and secondary endpoints such as central and peripheral pressures and pulse pressure (PP). Univariate and bivariate analyses were conducted using appropriate statistical tests.

Results

A comparative prospective observational study was completed on 58 patients (34 women and 24 men; mean age: 52.64 +/- 12.12 years) treated with anti-cancer chemotherapy agents. Our evaluation included a complete clinical exam, electrocardiogram, Doppler echocardiography, and applanation tonometry with arterial stiffness measurement using PWV. Patients presented significantly higher levels of carotid-femoral PWV, regardless of the chosen chemotherapy protocol, with no return to the initial level after one year of stopping treatment (p-value < 0.01). Moreover, this increase was more significant in patients with diabetes and hypertension and patients treated with monoclonal antibodies or intercalants.

Conclusion

This prospective study shows that chemotherapy patients have elevated arterial stiffness, emphasizing the need to assess PWV and monitor cardiovascular risk factors. PP measurement with PWV could improve risk management.

## Introduction

Cancer and cardiovascular disease (CVD) are the two significant causes of morbidity and mortality worldwide [1-5]. Cancer patients are vulnerable to many events that together make a person more susceptible to decreased cardiovascular functions, development of CVD, and ultimately to death [6-8]. Chemotherapy could lead to CVD directly through damages caused by the therapy itself or atherosclerosis induced by cancer treatment-related CV risk factors [9-12]. Cardiovascular co-morbidities like smoking, diabetes, and hypertension are frequently associated with numerous neoplastic diseases. In elderly patients with cancer, CVD is commonly associated with 20% of patients aged above 70 years.

Those newly diagnosed with cancer often have an associated clinical CVD or a higher risk of dying from a cardiovascular complication. CVD can precede cancer diagnosis and can also be a complication of cancer therapy [9,11,12]. To help understand the pathophysiology of CVD development, several important CVD risk factors, including hypertension, lifestyle, and age, were associated with observed changes in heart rate, blood pressure, and arterial structure [13,14]. Arterial stiffness is characterized by structural remodeling and functional changes in the arterial wall [15,16]. Thus, any altering condition would transmit damaging pulsatile flow through the circulatory system, affecting the long-term risk of cardiovascular disease morbidity and mortality [15-17]. This criterion can be estimated by measuring blood flow rate (pulse wave velocity [PWV]), which procures an augmentation index (AIx) to reflect the pulse wave from subsidiary stages in the arterial tree [18,19]. 

Anti-cancer chemotherapy is a prevalent treatment option for several types of cancer that, although effective in expanding cancer survival, is associated with a decline in cardiovascular health [8,10,11]. Therefore, the risk of cardiovascular disease increases in cancer survivors, especially those with a history of chemotherapy [10]. Thus, anticipative uncovering of cardiovascular disease in cancer patients experiencing anti-cancer treatment is decisive in improving long-term survival. Cardiovascular toxicity of anti-cancer treatments seems to be an additional risk factor in accelerating the process of vascular aging, which is expressed on the vascular level by the presence of fibrosis of the arterial wall and on the myocardial level by an alteration of relaxation, both contributing to the arterial stiffness causing damages [8,20-27]. Few studies have looked at vascular toxicity secondary to cancer chemotherapy, mainly through the study of PWV [28-32]. The type and frequency of cardiovascular complications encountered depend on the anti-mitotic agent used, its cumulative dosage, and the associations in which it is included (potentiating effect). Almost all studies on the side effects of chemotherapy concluded that whatever the product’s mechanism of action, it leads to endothelial dysfunction. Numerous studies have demonstrated that endothelial dysfunction is also involved in the pathophysiology of atherosclerosis-related cardiovascular diseases [33-36]. Our study is part of this recent research effort worldwide; thus, we would like to answer the question: do drugs used in oncology, mainly chemotherapy, increase arterial stiffness?

## Materials and methods

Study design

**Figure 1:**
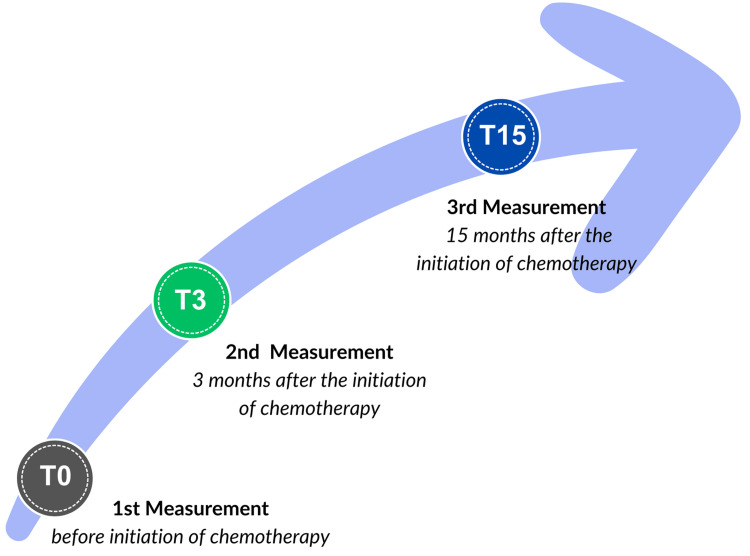
Follow-up time with intervals for measurements and examinations.

We conducted a prospective observational study approved by the Ethics Committee of CHU Mustapha University Hospital in Algiers, Algeria (authorization number: 3338, year: 2013). Our research aimed to assess arterial stiffness at three key time points: before cancer chemotherapy exposure (T0), three months after treatment initiation (T3), and 15 months post-treatment initiation (T15), as illustrated in Figure 1.

Patients treated for malignant tumors from various locations in the body (e.g., digestive, mammary) and subjected to various chemotherapy drug classes were followed up by the Department of Cardiology A2 of the Mustafa Bacha University Hospital of Algiers, Algeria. We recruited the patients referred by the Pierre and Marie Curie Medical Oncology Center of Algiers (CPMC) and the Bouzaréah Medical Oncology Center from November 2013 to June 2015. All patients provided written informed consent before enrollment.

Population study

In this investigation, a cohort of 58 patients diagnosed with neoplastic disease and undergoing diverse chemotherapeutic treatments were considered. Inclusion criteria comprised individuals 18 years or older who were initiating primary intravenous chemotherapy, possessing a performance status between 0-2, and not undergoing hormone therapy or anti-tumor immunotherapy. Exclusion criteria involved individuals with a history of previous chemotherapy or radiation therapy, cardiovascular events (including heart failure, angina pectoris, myocardial infarction, coronary revascularization, cardiac arrhythmias, significant valve disease, aortic aneurysm, and megadolicho artery), elevated C-reactive protein (CRP) levels (>10 mg/L), recent infectious or inflammatory diseases (or those undergoing treatment within the past two weeks), individuals on kidney dialysis, those with a significantly compromised general condition based on Karnofsky’s classification [37], and individuals classified as obese with a BMI exceeding 30 Kg/m2.

Cardiovascular evaluation

During the observation period, patients underwent a comprehensive cardiovascular evaluation at a scheduled time, following the selection criteria outlined in the study protocol. The diagnosis of high blood pressure was established based on systolic (SBP >140 mmHg) or diastolic (DBP >90 mmHg) levels or if the patient was under antihypertensive treatment. Diabetes was determined by a fasting blood sugar level greater than 1.26 g/L, an HbA1C 6.5%, or hypoglycemic drugs. Dyslipidemia was defined as total cholesterol > 1.9 g/L (6.5 mmol/L), LDL-cholesterol > 1.6 g/L (4.0 mmol/L), or HDL-cholesterol < 0.4 g/L (1.0 mmol/L) in men and < 0.46g/L (1.2 mmol/L) in women. Smoking status (packs/year), menopause (absence of menstruation > 12 months), and any episode of CVD history in the family were noted, and a complete clinical examination was conducted.

After at least five minutes of rest, hemodynamic measurements were taken on patients who abstained from caffeine and smoking for at least 16 hours before the arterial assessment. SBP, DBP, and heart rate were measured using a manual mercury sphygmomanometer (Spengler, France). Peripheral and central systolic/diastolic blood pressure (pSBP, pDBP, cSBP, cDBP), pulse pressure (PP), and PWV were measured using a validated system (SphygmoCor XCEL; AtCor Medical, Sydney, Australia) that applies the applanation tonometry and pertinent acquisition software for noninvasive recording and analysis of the arterial pulse. The carotid-femoral PWV calculation is known as: PWV = distance [m]/transit time [seconds].

The PWV value of 10 m/s was considered an objective marker of end-organ damage by the European Society of Cardiology (ESC), as stated in the expert consensus document on the measurement of aortic stiffness in daily practice using carotid-femoral PWV [38]. However, there is still no consensus on fixed PWV-threshold values, and arterial stiffness increases physiologically with aging. Thus, this threshold does not have the same prognostic value in a young subject as in an elderly one. European Heart Journal published "Determinants of pulse wave velocity in healthy people and in the presence of cardiovascular risk factors: establishing normal and reference values" [39]. The normal values of the population were used, that is, those measured in subjects without any CV risk factor published in 2010 (Table 1), in which there is no significant difference between men and women. Thus, any value beyond the 90th percentile is considered pathological.

**Table 1:** Distribution of pulse wave velocity (m/s) according to the age category in the population of the normal value (1455 subjects) SD, standard deviation; 10 pc, the upper limit of the 10th percentile; 90 pc, the lower limit of the 90th percentile.

Age category (years)	Mean (±2 SD)	Median (10–90 pc)
<30	6.2 (4.7–7.6)	6.1 (5.3–7.1)
30–39	6.5 (3.8–9.2)	6.4 (5.2–8.0)
40–49	7.2 (4.6–9.8)	6.9 (5.9–8.6)
50–59	8.3 (4.5–12.1)	8.1 (6.3–10.0)
60–69	10.3 (5.5–15.0)	9.7 (7.9–13.1)
+70	10.9 (5.5–16.3)	10.6 (8.0–14.6)

All patients had a baseline electrocardiogram (using a Cardioline ECG device [Cardioline SpA, Linz, Austria]). Doppler echocardiographic studies were performed by the same operator each time, on the same day as the measurements by applanation tonometry, using a GE HealthCare VIVID 7 device (GE HealthCare, Chicago, IL) equipped with a 4.0 MHz frequency probe with a second harmonic, a two-dimensional (2D) mode, a time mode movement (TM), a Doppler mode (pulsed, continuous and color) and software for studying the longitudinal systolic function of the left ventricle (2D strain). Myocardial deformation indices (GLS: global longitudinal strain) were measured in all patients.

Chemotherapy

Medical professionals treated the patients with various drugs and protocols to combat different types of cancer, such as intercalants (anthracyclines), alkylating agents, antimetabolites, anti-microtubule agents, and monoclonal antibodies. Combining chemotherapy agents is commonly used for various cancer types and is applied to multiple cancer patients. Furthermore, studies that evaluated cardiotoxicity previously found that cancer patients undergoing treatment were administered multiple chemotherapy agents [23,8].

Statistical analysis

We performed all statistical analyses using R statistical software (R Foundation for Statistical Computing, Vienna, Austria) [40].

We used the “sample size” package [41] to determine the sample size, which was 52 patients who met the inclusion and exclusion criteria, for the comparison of two means for paired series assuming expected values of two meters/second, a standard deviation of five meters/second, an alpha risk of 5%, a power of 80%, and equality of variance. We set statistical significance at p=0.05.

We developed a standardized structured questionnaire to collect data on cardiovascular risk factors, using data reported in major international studies (INTERHEART, Framingham, and WISE) [42,43], and international recommendations [4].

The primary endpoint directly assessed the increase in arterial stiffness by measuring PWV using applanation tonometry of 2 meters/second. The secondary endpoints measured pSBP, pDBP, cSBP, cDBP, and PP. We used the same device to study and estimate these three parameters during the same examination time.

For the univariate analysis, we presented the qualitative variables by frequency and rank (quartiles and medians, min and max values) and the quantitative variables by their mean and class. For the bi-variate analysis, we used McNemar’s chi-squared test for paired series to compare qualitative variables, while Fisher’s exact test was used for small groups. We used the Student’s test for paired series to compare quantitative variables, and we reported the values of the variables in a 95% confidence interval (allowing a 5% risk of alpha error).

## Results

General population characteristics

In this study, we analyzed 58 out of 174 participants, with a high loss rate of 60%.

Table 2 shows the demographics and clinical characteristics of the patients. The mean age of both sexes was around the fifties, with no significant difference between men and women (34 women and 24 men; mean age: 52.64 ± 12.12 years, range: 18-76 years, P = 0.19), and almost one-third of the female population was menopausal. The population had no significant overweight (mean BMI: 25.85 ± 4.58 kg/m2, P = 0.12), and almost a third of the patients were either diabetic (n = 13, P= 0.43) or hypertensive (n = 17, P = 0.36), well-stabilized and undergoing treatment at enrollment.

**Table 2:** Patient baseline characteristics (demographics and clinics). n: number of patients, BMI: Body Mass Index.

	Women (n=34); 58.62 %	Men (n=24); 41.38%	All (n=58); 100%	P-value
Age (years)				0.19
Mean	50.88	55.13	52.64	
SD	11.87	12.28	12.12	
Median	50	56	54	
Range	18-76	33-76	18-76	
Weight (kg)				0.61
Mean	70.88	69.25	70.21	
SD	13.35	10.86	12.31	
Median	69	70	70	
Range	46-106	42-90	42-106	
Height (cm)				0.0047
Mean	160.83	171.04	165.05	
SD	5.93	8.05	8.50	
Median	162	172	165	
Range	147-173	172	165	
BMI (kg/m2)				0.12
Mean	27.41	23.64	25.85	
SD	4.86	3.08	4.58	
Median	26.94	24.31	25.67	
Range	17.97-40.39	17.97-40.39	17.97-40.39	
Diabetes (n)	6 (17.65 %)	7 (29.17 %)	13 (22.41 %)	0.43
Hypertension (n)	12 (35.29 %)	5 (20.83 %)	17 (29.31 %)	0.36

Table 3 comprehensively summarizes the patients' cancer characteristics and treatments. The study cohort consisted of individuals diagnosed with breast cancer (n=18, predominantly women), colic cancer (n=11, predominantly men), ear-nose-larynx cancer (n=9), digestive cancer (n=8, predominantly men), genital cancer (n=8, predominantly men), and other types (n=4). Notably, nearly half of the participants were in the metastatic stage (n=25), with a notable occurrence in the liver (28% liver only, 16% liver and bone). The distribution of neoplasia types demonstrated statistical significance (P = 0.003), while the distribution of metastatic localization did not reach statistical significance (P = 0.42).

**Table 3:** Patients’ cancer details. n: number of patients.

	Women (n=34)	Men (n=24)	All (n=58)	P-value
Neoplasia type (n)				0.003
Breast	16	2	18 (31%)	
Colon	6	5	11 (19%)	
Nose-ear-larynx	5	4	9 (15%)	
Digestive	2	6	8 (14%)	
Genital	2	6	8 (14%)	
Other	3	1	4 (7%)	
Metastases (n)				0.42
yes	13	12	25 (43.10%)	
no	21	12	33 (56.90%)	
Metastatic localization (n)				0.39
Liver	3	4	7 (28%)	
Lung + lymph node	2	3	5 (20%)	
Bone	2	2	4 (16%)	
Liver + bone	3	1	4 (16%)	
Lymph node	2	1	3 (12%)	
Cerebral	2	0	2 (8%)	
Total number of patients	13	12	25	

In the general population (n = 58), Table 4 illustrates the distribution of various chemotherapeutic agents administered to the patients. These included alkylating agents (n = 38, primarily platinum salts), antimetabolites (n = 33, primarily 5-FU), monoclonal antibodies (n = 33, mainly bevacizumab for metastatic colonic and rectal neoplasia, and trastuzumab for breast cancer), anti-microtubules (n = 28, mainly paclitaxel), and anthracyclines (n = 23, primarily intercalants). The cumulative average doses of the five antineoplastic agents did not show statistical significance during the 15-month observation period (P > 0.05). This trend persisted when considering the cumulative doses administered over the entire treatment duration.

**Table 4:** Distribution according to different administered chemotherapeutic agents

Drug class	Treated with	Not treated with
Alklatying agents	38 (65.5%)	20 (34.5%)
Antimetabolites	33 (56.9%)	25 (43.1%)
Antimicrotubules	28 (48.3%)	30 (51.7%)
Monoclonal agents	33 (56.9%)	25 (43.1%)
Anthracyclines	23 (39.6%)	35 (60.4%)

Vascular stiffness parameters

**Figure 2:**
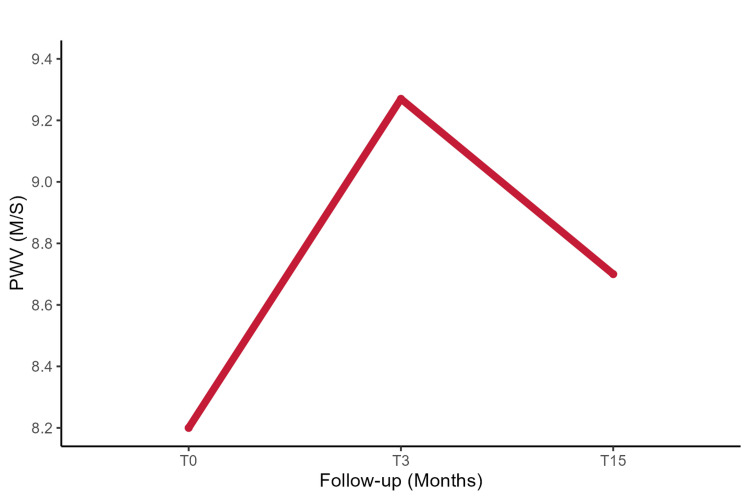
Changes in the mean PWV during the follow-up period. PWV: Pulse wave velocity

The temporal changes in PWV before and after chemotherapy demonstrate a significant and persistent increase during antineoplastic treatment, with no restoration to the baseline (T0) even after one year following therapy discontinuation. Importantly, a highly significant difference in PWV is observed between the initial values and those measured at T3, with a p-value of less than 0.0001. This significant variation is maintained at the T15 follow-up, as indicated in Figure 2, with a statistically significant p-value of 0.01.

**Figure 3:**
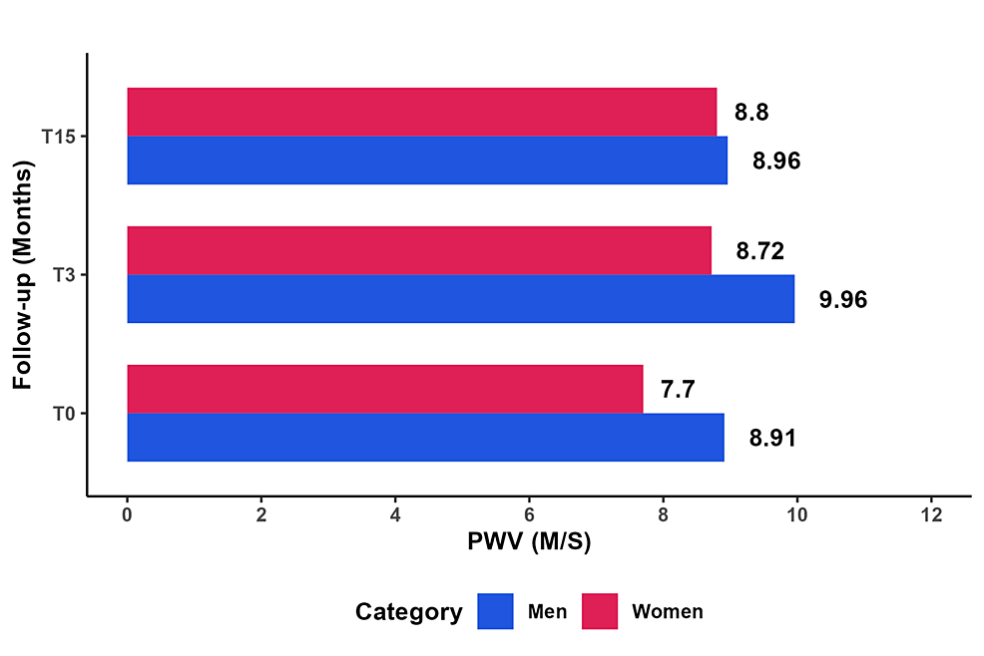
Comparative analysis of the average PWV by sex PWV: Pulse wave velocity

The study findings indicated a higher PWV in men compared to women, and this discrepancy persisted consistently throughout the entire duration of the study, as illustrated in Figure 3. Although both sexes experienced an increase in PWV without a significant difference, it is noteworthy that men exhibited a return to baseline levels (with values at T0 and T15 being quite similar), while women did not demonstrate the same pattern, as depicted in Figure 3.

There were no significant changes in peripheral and central pressures or AIx, except for the PP between T0 and T15, which went in the same direction as PWV (P=0.013), as shown in Table 5. Sex did not significantly influence these parameters (P>0.05).

**Table 5:** Temporal analysis of the parameters determining arterial stiffness pSBP: peripheral systolic blood pressure, pDBP: peripheral diastolic blood pressure; cDBP: central diastolic blood pressure; cSBP: central systolic blood pressure;  PP: pulse pressure; AIx: augmentation index

	T0 - T3			T3 - T15			T0 - T15		
Variable	Mean	SD	P	Mean	SD	P	Mean	SD	P
pSBP	-0.15	14.63	0.93	3.06	16.48	0.16	2.91	17.55	0.21
pDBP	-1.08	8.66	0.34	-0.82	9.76	0.52	-1.91	11.15	0.19
cSBP	0.2	13.33	0.9	2.75	13.66	0.12	2.96	16.48	0.17
cDBP	-0.75	8.82	0.51	-0.82	10.23	0.54	-1.58	11.38	0.29
PP	1.46	11.81	0.34	2.08	10.86	0.14	3.55	10.64	0.013
AIx	0.53	11.12	0.71	7.67	41.72	0.16	8.2	40.42	0.12

Correlation with risk factors

All hypertensive and diabetic patients exhibited an elevation in PWV following chemotherapy, which remained elevated and did not revert to baseline, regardless of the degree of PWV fluctuations. Hypertensive and diabetic individuals demonstrated higher PWV levels than normotensive and non-diabetic individuals. Significantly, we observed that the increase in PWV was proportional to the baseline value in both groups, with no significant differences.

**Figure 4:**
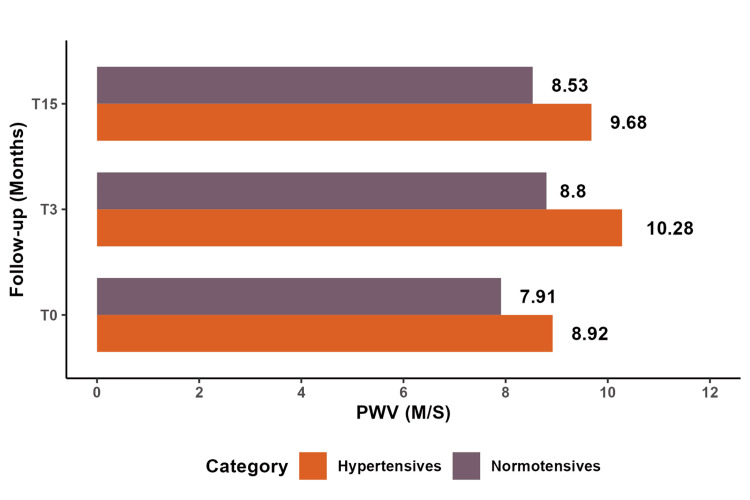
Comparative study of mean PWV between hypertensive and normotensive patients. PWV: pulse wave velocity

Figure 4 demonstrates a significant increase in PWV among hypertensive patients compared to normotensive patients at both T3 (P=0.05) and T15 (P=0.03). PWV did not return to baseline in either group.

Table 6 presents a comparison of arterial stiffness parameters between normotensive and hypertensive patients at T3 and T15. At T3, hypertensive patients exhibited higher mean values of peripheral systolic blood pressure (pSBP) and central systolic blood pressure (cSBP) compared to normotensive patients, with statistically significant differences (pSBP: P=0.025, cSBP: P=0.023). At T15, hypertensive patients had significantly higher PP) compared to normotensive patients (P=0.03). PWV values exhibited a trend towards significance at T3 (P = 0.05) and T15 (P = 0.03).

**Table 6:** Comparing arterial stiffness parameters in normotensive and hypertensive patients at T3 and T15. pSBP: peripheral systolic blood pressure, pDBP: peripheral diastolic blood pressure; cDBP: central diastolic blood pressure; cSBP: central systolic blood pressure; PP: pulse pressure; PWV: pulse wave velocity

	Normotensives		Hypertensives		
Variables	Mean	SD	Mean	SD	P value
pSBP (T3)	121.2	13.89	132.1	16.58	0.025
pSBP (T15)	124.8	11.84	134	19.86	0.08
cSBP (T3)	111.1	11.82	120.5	14.3	0.023
cSBP (T15)	76.1	8.96	78.82	8.84	0.29
PP (T3)	35.41	9.12	40.71	11.23	0.26
PP (T15)	36.93	7.05	44.18	12.72	0.03
PWV (T3)	8.8	1.88	10.28	2.75	0.05
PWV (T15)	8.53	1.62	9.68	1.86	0.03

**Figure 5:**
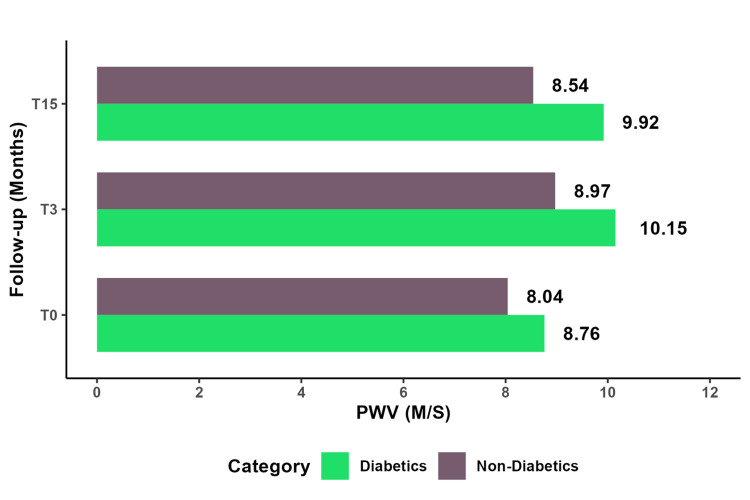
Comparative study of mean PWV between diabetic and non-diabetic patients. PWV: pulse wave velocity

Similar variations were observed in diabetic patients as in hypertensive patients. At the same assessment, there was a significant increase in PWV among diabetic individuals compared to non-diabetic individuals (T3: P=0.05; T15: P=0.022), as seen in Figure 5 and Table 7. No notable variations were observed in the other parameters.

**Table 7:** PWV means in diabetics and non-diabetic patients at T3 and T15. PWV: pulse wave velocity

	Non-Diabetics		Diabetics		
Variables	Mean	SD	Mean	SD	P-value
pSBP (T3)	123.5	15.8	127.6	14.03	0.37
pSBP (T15)	126.3	16.2	131.7	9.52	0.13
cSBP (T3)	76.27	9.71	77.15	7.9	0.73
cSBP (T15)	75.2	8.3	77.15	8.16	0.45
PP (T3)	36.98	10.7	36.92	7.32	0.98
PP (T15)	38.42	10.3	41.23	6.2	0.23
PWV (T3)	8.97	2.3	10.15	1.69	0.05
PWV(T15)	8.54	1.59	9.92	1.89	0.022

Association of arterial stiffness variation based on drug class

Alkylating Agents

**Figure 6:**
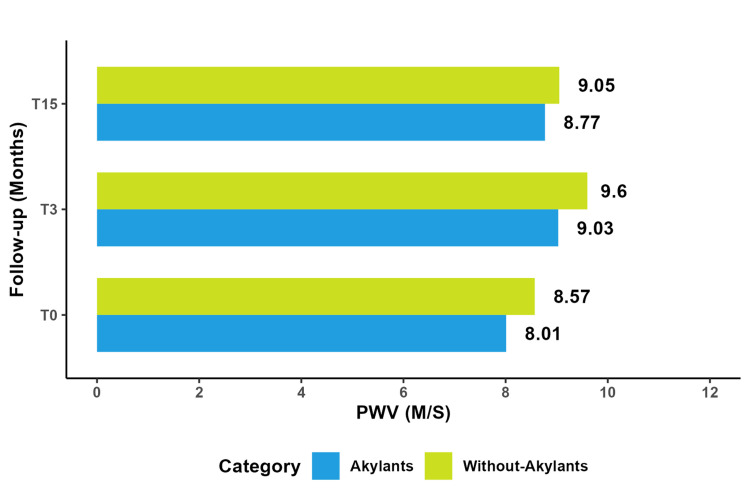
Comparative study of mean PWV with and without alkylating agents. PWV: pulse wave velocity

During the same examination, we conducted a comparative study of PWV between two groups: one receiving alkylating agents and the other not receiving them. The results showed no significant difference in PWV between the two groups (SBP: P = 0.56, cSBP: P = 0.31, pDBP: P = 0.61, cDBP: P = 0.24, and PP: P = 0.42). Both groups experienced an increase in PWV, with a rise in mean PWV. However, this increase was not statistically significant between the groups (P = 0.62). Furthermore, neither of the groups returned to baseline PWV levels after the specified period (Figure 6).

Antimetabolites

**Figure 7:**
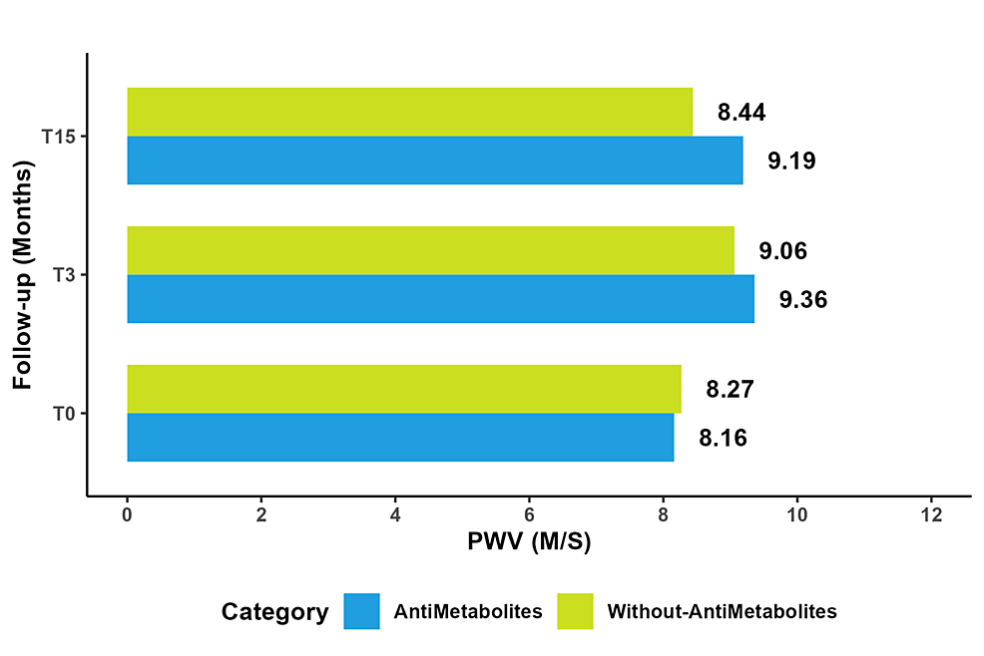
Comparative study of mean PWV with and without antimetabolites. PWV: pulse wave velocity

The comparative study between patients who received antimetabolites and those who did not, within the same examination, did not reveal a significant difference. Both groups experienced an increase in PWV, but no statistically significant variation was observed between the "with" and "without" groups (P = 0.12). It should be noted that patients who received antimetabolites did not return to baseline levels (Figure 7).

Anti-microtubules

**Figure 8:**
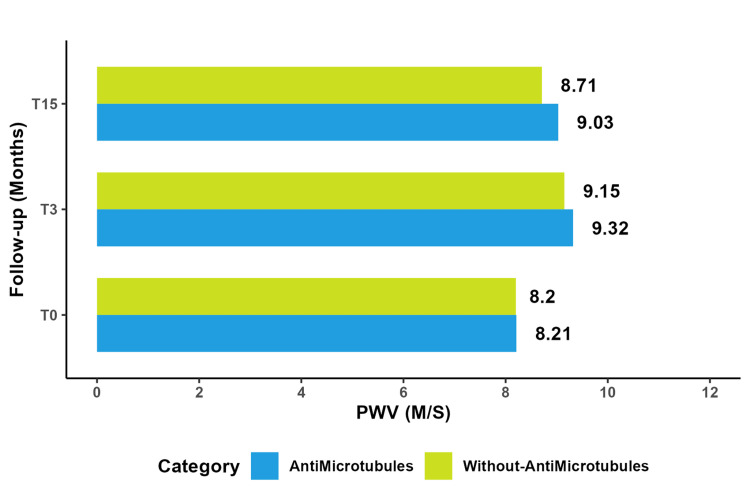
Comparative study of mean PWV with and without anti-microtubules. PWV: pulse wave velocity

In a comparative study, we observed that patients who received anti-microtubule treatment had significantly higher pSBP and cSBP compared to those who did not receive the treatment over the interval (T0-T3, respectively, P =0.025; P=0.023). Additionally, the difference in pDBP and cDBP was more pronounced in the T15 examination group. Although PWV increased in both groups, there were no significant differences between them, and the values did not return to baseline after one year (Figure 8).

Intercalants

**Figure 9:**
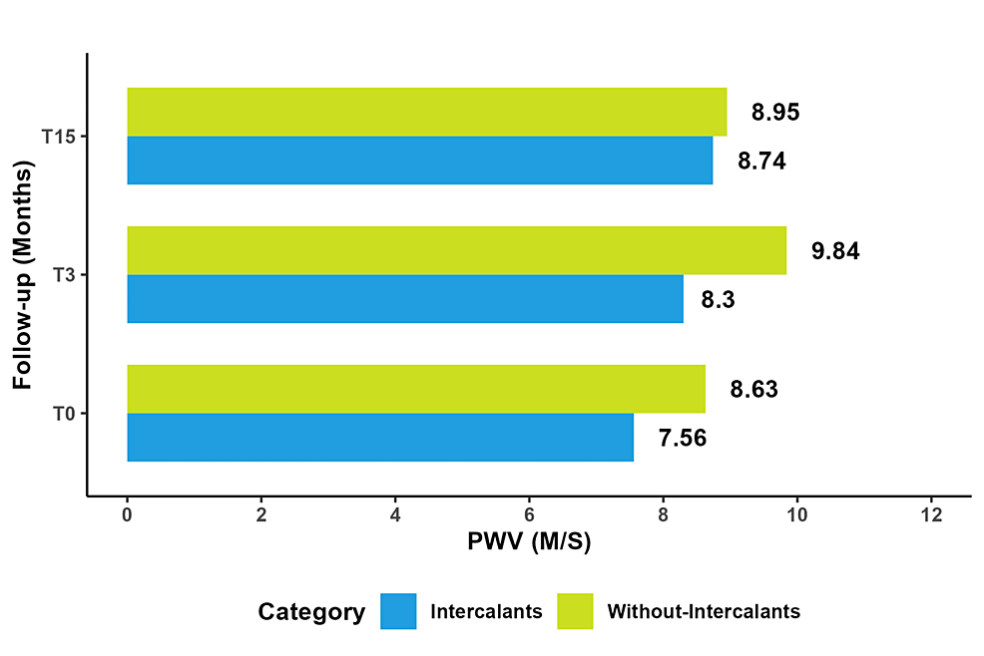
Comparative study of mean PWV with and without intercalants. PWV: pulse wave velocity

A comparative analysis between patients who received intercalating agents and those who did not reveal a significant difference at T0 and T3 (P=0.01 and P=0.004, respectively). The PWV increased in both groups, but patients treated with intercalating agents demonstrated a significant and early increase in PWV between T0 and T3 compared to those who did not receive them (P=0.025). There were no notable differences in other parameters. Furthermore, the increase in PWV was significantly higher in patients treated with intercalating agents, independent of angiogenic factors. Notably, during chemotherapy, patients receiving intercalating agents exhibited a marked elevation in PWV that persisted over time, as illustrated in Figure 9.

Monoclonal Antibodies

The analysis comparing patients who received monoclonal antibodies to those who did not revealed significant differences at T0 and T3. Additionally, at T15, a noteworthy advantage was observed in favor of patients who received monoclonal antibodies (P=0.0015). Both groups experienced increased PWV, with a significantly more significant increase among patients who received monoclonal antibodies (T0-T3) (P=0.03). This difference in PWV increase also persisted when comparing the pre-chemotherapy state to the post-chemotherapy state (T0-T15) (P=0.05).

**Figure 10:**
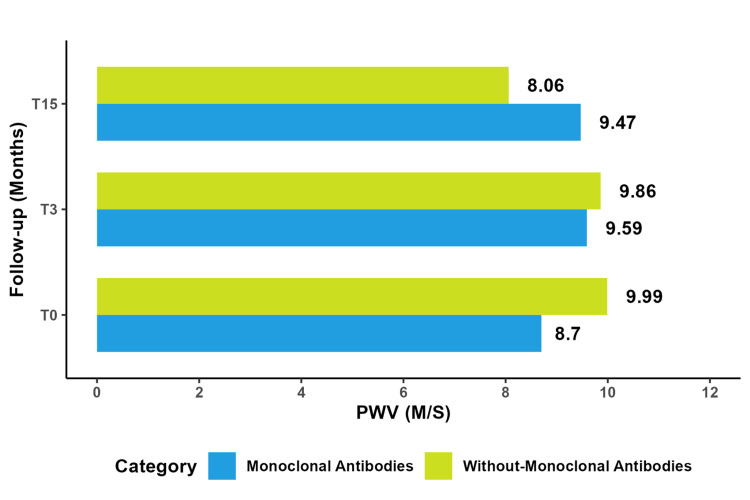
Comparative study of mean PWV with and without monoclonal antibodies. PWV: pulse wave velocity

Our study yielded positive results regarding our primary endpoint, which focused on measuring PWV using applanation tonometry at a speed of 2 meters per second. We observed a significant increase in PWV (p-value < 0.0001) following the administration of antineoplastic chemotherapy. However, the magnitude of this increase varied and was not statistically significant during the period between the end of treatment T3 and the follow-up examination at T15. It is noteworthy that PWV did not return to its initial level between the measurements taken before chemotherapy initiation (T0) and the follow-up at the end of chemotherapy (T15). Furthermore, the increase in PWV was particularly significant (p-value = 0.011) among patients with hypertension, diabetes, and those receiving monoclonal antibodies. We did not observe significant changes in peripheral systolic blood pressure, central systolic pressure, or AIx. However, PP exhibited a significant increase (p-value = 0.01) between the pre-chemotherapy measurement and the one conducted at T15, following the same trend as PWV (Figure 10).

About Cumulative Doses

The analysis of the average cumulative doses revealed exciting findings among the different groups based on the use of specific chemotherapy agents. The "anti-metabolites" group showed the highest average cumulative dose of 38600, but no significant differences were observed compared to other groups, with a p-value of 0.28. Conversely, the "alkylating agents" group had the lowest average cumulative dose of 3529, and there were no significant differences in doses among the groups, with a relatively lower p-value of 0.13. The "anthracyclines," "monoclonal AC," and "microtubules" groups had average cumulative doses of 1718, 2756, and 674.1, respectively, and none of these groups displayed statistically significant differences in doses, with p-values of 0.97, 0.77, and 0.87, respectively.

In summary, Table 8 provides an overview of the distribution of average cumulative doses for each chemotherapy drug category, showing no statistically significant differences in doses among patients.

**Table 8:** The distribution of average cumulative doses and corresponding standard deviations (SD) for various chemotherapy drug categories utilized during the treatment.

Chemotherapy Drug Category	Average Cumulative Doses	Standard Deviation (SD)	p-value
Alkylating agents	3529	5993	0.13
Anti-metabolites	38600	106200	0.28
Anthracyclines	1718	5707	0.97
Monoclonal AC	2756	3072	0.77
Microtubules	674.1	1133	0.87

## Discussion

This study revealed a significant increase in post-chemotherapy PWV values across all chemotherapy regimens (P < 0.0001). Among secondary endpoints, only PP exhibited a noteworthy increase (P=0.01), underscoring its potential as an indicator of arterial stiffness. These findings emphasize the importance of monitoring PWV and PP as valuable metrics in assessing cardiovascular health in post-chemotherapy patients. Further research may elucidate the clinical implications of these findings and refine their use in patient care.

The rise in arterial stiffness indices immediately at the end of chemotherapy (T3) indicates an acute process rather than a chronic one, as seen in previous studies in metastatic malignancies and anthracycline chemotherapy [44,45]. This acute vascular toxicity could also be associated with microthrombi formation and perturbed blood flow, which are prevalent in malignancies [46,47]. At the same time, AIx, this indirect measurement of arterial stiffness, was not significant in contrast to various studies [48-50]. AIx is a composite parameter that mainly influences sex, age, height, and heart rate [51,52].

There was a significant rise in PWV levels post-chemotherapy in hypertensive and diabetic patients. Unlike a steady blood pressure rise over time in the hypertensive group [44,45], we observed a notable difference between normotensive and hypertensive patients at T3. This suggests that heightened vasoconstriction might be more pronounced in the hypertensive group at the beginning of treatment, specifically during T3. However, the central and peripheral blood pressures with non-significant changes could probably be explained by the early usage of effective antihypertensive treatment, particularly in cases of iatrogenic hypertension [53,54].

Our study revealed that monoclonal antibodies and intercalants significantly increased PWV levels (P=0.025, P=0.03), mirroring findings in a study on breast cancer patients who underwent anthracycline trastuzumab-based therapy. What increased aortic stiffness put them at higher risk for future cardiovascular events [55]. However, we also observed that PWV continued to increase despite stopping intercalant treatments. 

Various agents can affect arterial stiffness through diverse mechanisms. Bevazicumab, ramucirumab, and rituximab are known to cause hypertension [56]. Additionally, monoclonal antibodies targeting inflammation pathways have been shown to lessen cardiovascular episodes despite inflammation increasing arterial stiffness [57-59]. 

Despite the recognized cardiovascular risks associated with alkylating agents, antimetabolites, and anti-microtubule chemotherapies supported by several studies, including those by Soultati et al. and Parr et al. [11,29], our study delivered a different outcome. In contrast to prior research [53, 60-62], our findings showed no significant rise in aortic stiffness (PWV) among patients undergoing treatments with these agents (with respective p-values of 0.13, 0.28, and 0.87). This discrepancy prompted a follow-up assessment of the research data to thoroughly investigate and address the possible reasons behind the observed divergence.

It was difficult to determine the post-chemotherapy reversibility in our work with certainty because most patients maintained their anti-hypertensive treatment with random compliance. Additionally, the statistical analysis relied on PWV at 2 meters/second, which is a relatively high value considering that an increase of 1 meter/second equals arterial aging of 10 years [39]. Therefore, we carefully monitored our patients as the rigidity of the vessels indicated significant aging of the arteries. Good management improved cardiovascular outcomes, particularly in young patients with better oncological prognoses.

The present study supports the need for specific vascular monitoring. Developing evidence-based guidelines for managing and monitoring vascular damage requires addressing the knowledge disparity identified in these findings [63-65]. A critical outcome of this study is that arterial stiffness, as measured by PWV, should be considered part of recommended care for at-risk patients because it is simple, non-invasive, cost-efficient, and reproducible. Ideally, specialists should collaborate to treat and correct cardiovascular risk factors before initiating antineoplastic treatment, which would greatly benefit the patient.

While our sampling is commendable, it is essential to acknowledge certain critiques. The notable heterogeneity in both sampling and the mechanisms of action of the utilized antineoplastic drugs on the arterial walls raises concerns. Additionally, it is prudent to consider addressing the potential for a more extended follow-up period to capture late results on chemotherapy-induced arterial stiffening. Importantly, the main limitation of our study is the deliberate absence of a control group, a decision made due to ethical considerations.

## Conclusions

Our study identifies a consistent elevation in arterial stiffness, measured by PWV, across diverse chemotherapy types without observed reversal. Despite not currently recognized as a cardiovascular disease marker, a parallel increase in pulse pressure was noted. While examinations of peripheral and central blood pressure and the AIx were inconclusive, the study strongly implies a link between oncological treatment, particularly monoclonal antibodies (bevacizumab [Avastin], trastuzumab [Herceptin]) and PWV variations. These changes in PWV appear independent of blood pressure levels, irrespective of pre-existing hypertension or antineoplastic medication usage. The importance of early identification and consistent care for high-risk individuals in chemotherapy patients is emphasized in this study by assessing pulse pressure and cardiovascular risk factors. Combining pulse pressure measurement with PWV assessment can potentially improve risk stratification and management strategies, underscoring the benefits of anti-cancer chemotherapy and targeted therapy. Future research should focus on optimizing these findings and exploring potential interventions.
